# Phylogenetic and structural analysis of the phospholipase A2 gene family in vertebrates

**DOI:** 10.3892/ijmm.2014.2047

**Published:** 2014-12-23

**Authors:** QI HUANG, YUAN WU, CHAO QIN, WENWU HE, XING WEI

**Affiliations:** 1 Department of Neurology, First Affiliated Hospital, Guangxi Medical University, Nanning, Guangxi, P.R. China; 2 Department of Cardiothoracic Surgery, Nanchong Central Hospital, The Second Clinical College of North Sichuan Medical College, Nanchong, Sichuan, P.R. China

**Keywords:** phospholipase A2, Bayesian information criterion, phylogenetic analysis, positive selection sites

## Abstract

The phospholipase A (PLA)2 family is the most complex gene family of phospholipases and plays a crucial role in a number of physiological activities. However, the phylogenetic background of the PLA2 gene family and the amino acid residues of the PLA2G7 gene following positive selection gene remain undetermined. In this study, we downloaded 49 genomic data sets of PLA from different species, including the human, house mouse, Norway rat, pig, dog, chicken, cattle, African clawed frog, Sumatran orangutan and the zebrafish species. Phylogenetic relationships were determined using the neighbor-joining (NJ), minimum evolution (ME) and maximum parsimony (MP) methods, as well as the Bayesian information criterion. The results were then presented as phylogenetic trees. Positive selection sites were detected using site, branch and branch-site models. These methods led us to the following assumptions: i) closer lineages were observed between PLA2G16 and PLA2G6, PLA2G7 and PLA2G4, PLA2G3 and PLA2G12, as well as among PLA2G10, PLA2G5 and PLA2G15; ii) PLA2G5 appeared to be the origin of the PLA2 family, and PLA2G7 was one of the most evolutionarily distant PLA2 proteins; iii) 16 positive-selection sites were detected and were marked in the PLA2G7 protein sequence as 327D, 257Q, 276G, 34s, 66G, 67C, 319S, 28N, 50S, 54T, 58R, 75T, 88Q, 92R, 179H and 191K.

## Introduction

The phospholipase gene family encodes enzymes that hydrolyze phospholipids into fatty acids and other micromolecules. This gene family is classified into four major classes, namely, phospholipase PLA, PLB, PLC and PLD (1), based on the types of catalytic reaction of phospholipids. The majority of coding enzymes play crucial roles in lipid metabolism (2), cell proliferation (3), muscle contraction (4-6) and in the inflammation process (5). The PLA class includes two subfamilies, namely PLA1 and PLA2. PLA1 cleaves the SN-1 acyl chain and is a major component of snake venom (7,8), whereas PLA2 cleaves the SN-2 acyl chain and releases arachidonic acid, which mediates anti-inflammatory and inflammatory responses (9). The PLA2 gene family is divided into nine groups based on their function: PLA2G3, PLA2G4, PLA2G5, PLA2G6, PLA2G7, PLA2G10, PLA2G12, PLA2G15 and PLA2G16 (10). The coding enzymes of these genes are important in platelet activity (11,12) and B-cell activity (13,14). The dysfunction of one or more of these genes leads to stroke (14) and other neurological diseases (15–17). However, all the functions of these genes have not yet been fully elucidated.

A number of studies have focused on the association between the PLA2 gene family and various physiological and pathological conditions. The results of a clinical trial demonstrated that high levels of sPLA2 mass in the circulation are not associated with a high risk of cardiovascular disease (18), which is not consistent with some earlier basal medical studies (11). Apart from potential flaws during study design, the differences in the results obtained may be attributed to genetic alterations reflecting a greater sensitivity to cardiovascular risk. In addition, certain studies have performed phylogenetic analyses (19). However, the available information is still insufficient partly due to methodological limitations and gene data inclusion criteria.

In the present study, we analyzed the functions and phylogenetic background of the PLA2 gene family. We aimed to firstly determine the phylogenetic background of the PLA2 gene family in vertebrates using the neighbor-joining (NJ), minimum evolution (ME) and maximum parsimony (MP) methods, as well as the Bayesian information criterion, and secondly, to detect the positive selection sites of the PLA2 gene family to define the structure and biological activity of the gene by site-directed mutagenesis, which may provide possible therapeutic targets. The data presented in this study provide insight into the phylogenetic relationships and functional differentiation of the phospholipase and PLA2 gene families.

## Materials and methods

### Data collection

We searched and downloaded natural and intact amino acid and gene sequences of the phospholipase and PLA2 families from the NCBI database (http://www.ncbi.nlm.nih.gov/gene/). These sequences included human, house mouse, Norway rat, pig, dog, chicken, cattle, African clawed frog, Sumatran orangutan and zebrafish sequences.

### Sequence alignment

We used the EBI web tool, MUSCLE (20), to align the sequences of the phospholipase and PLA2 family proteins. Rearranged gene sequences were generated according to the new amino acid alignment. The results of the amino acid alignment were placed in an aligned CDS fasta file using the EMBL web tool, PAL2NAL (21) (http://www.bork.embl.de/pal2nal/), which can form multiple codon alignments from matching amino acid sequences. The format was converted with the use of MEGA4.0. software (22).

### Phylogenetic analysis

The full alignment of sequences was used for the phylogenetic analysis. Akaike Information Criterion in PAUP^*^ version 4.0 (23) was applied to evaluate the most appropriate model of amino acid substitution for early tree-building analyses. ML optimizations and distance methods were valued by the PhyML program in PAUP^*^ version 4.0 (24). The most appreciated evolution type, GTR+I+G, was computed for the PLA2 gene family using Modeltest version 3.7 (25). Phylogenetic trees were reconstructed using the Bayesian method from the DNA alignment with the use of MrBayes version 3.1.2 software (26,27) according to the best-fit predictive model. The parameters for tree generation were as follows: 2×10^6^ generations of the PLA2 gene family were included with sampling every 1,000 generations, and with four chains (three cold, one heated); the first 250,000 generations (250 trees) were discarded from every run for the two families (phospholipase and PLA2). Analyses with the NJ, ME and MP methods were performed using MEGA4.0. software (22).

### Estimation of positive selection sites

**Figure 1:**
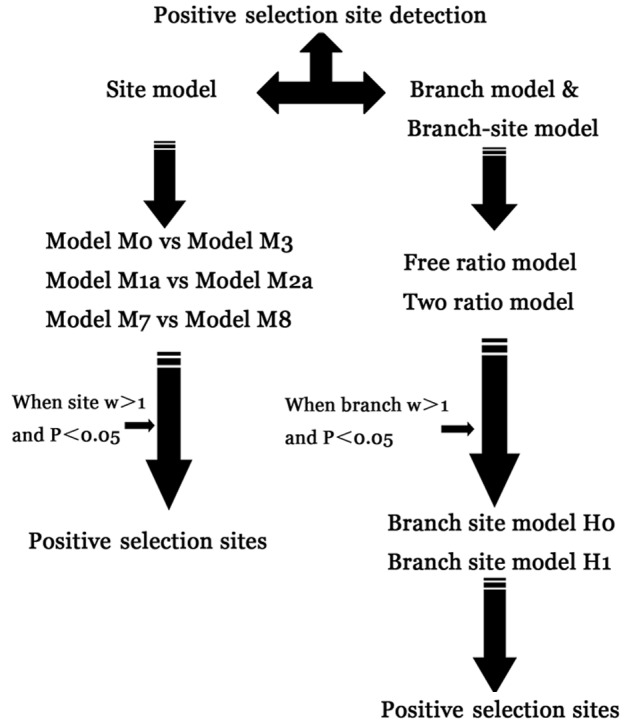
Flow of positive selective sites detection using hte site model, branch model and branch-site models.

Selective pressures of HA and NA genes were detected by CODEML in the PAML package version 4.4 (28). Three codon-based likelihood methods were run as branch, site and branch-site models. P<0.05 was used to determine whether or not the alternative hypothesis was significant. In these analyses, ML estimates of the selection pressure were based on the ratio dN/dS (ω), where dN and dS are the non-synonymous and synonymous substitution rates, respectively, which vary across codons; the probability of each codon being under positive selection was estimated. Positive selection sites can occur in very short episodes or on only a few sites during the evolution of duplicated genes when ω >1 (29). All alignments resulted from the PAL2NAL web tool. The parameter estimates (ω) and likelihood scores were calculated for three pairs of models: M0 (one ratio) vs. M3 (discrete); M1a (nearly neutral) vs. M2a (positive selection); and M7 (β) vs. M8 (β + ω). The likelihood ratio test (LRT) was used to compare the fit to the data of two nested models, assuming that twice the log likelihood difference between the two models (2ΔL) follows a χ^2^ distribution with a number of degrees of freedom equal to the difference in the number of free parameters (30). Naive empirical Bayes and empirical Bayes selection criteria implemented in PAML4 were used to identify sites under positive selection or relaxed purifying selection in the foreground group with significant LRTs. Each branch group was also labeled as a foreground group. The flow of positive selective site detection is presented in Fig. 1.

### Protein structure analysis and positive selection site marking

The protein sequence liner and 3D structure of PLA2 based on PLA2G7_Homo were created by the online tool, PredictProtein (31) (www.predictprotein. org), and I-TASSER (32–34) (http://zhanglab.ccmb.med.umich.edu/I-TASSER/). Functional areas were marked in Figs. 3 and 4.

## Results

### Phylogenetic analysis of PLA2 gene family in vertebrates

**Figure 2:**
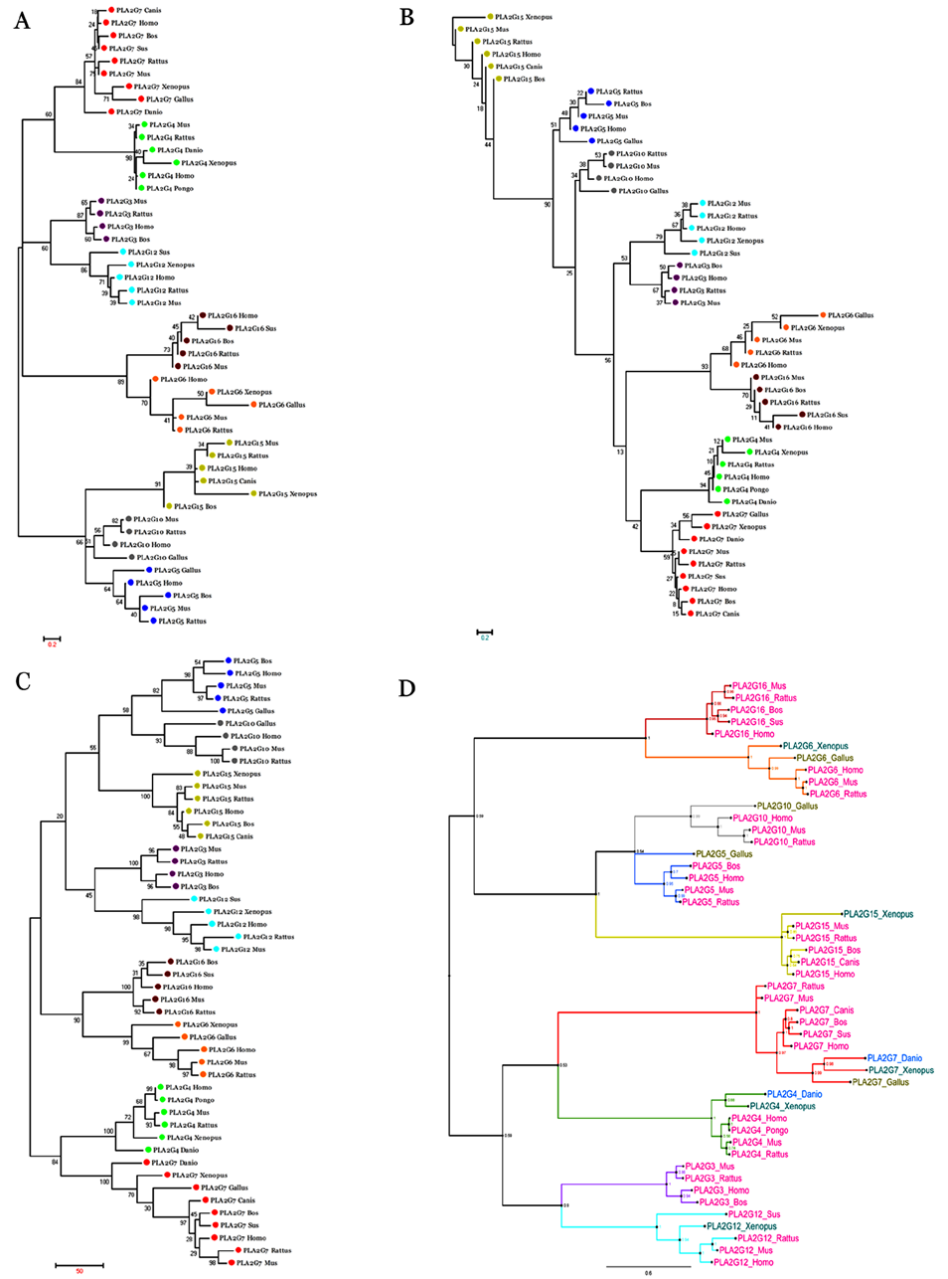
Phylogenetic trees of the PLA2 gene in vertebrates. (A) Phylogenetic tree produced using the NJ method; (B) phylogenetic tree produced using the ME method; (C) phylogenetic tree produced using the MP method. Genes with a crimson disc belong to the PLA2G16 group; genes with an orange disc belong to the PLA2G6 group; genes with a grey disc belong to the PLA2G10 group; genes with a dark blue disc belong to the PLA2G10 group; genes with a yellow disc belong to the PLA2G15 group; genes with a red disc belong to the PLA2G7 group; genes with a green disc belong to the PLA2G4 group; genes with a purple disc belong to the PLA2G3 group; genes with a light blue disc belong to the PLA2G12 group. (D) Phylogenetic tree of the PLA2 gene in vertebrates produced using the Bayesian method. Genes with a crimson branch belong to the PLA2G16 group; genes with an orange branch belong to the PLA2G6 group; genes with a grey branch belong to the PLA2G10 group; genes with a dark blue branch belong to the PLA2G10 group; genes with a yellow branch belong to the PLA2G15 group; genes with a red branch belong to the PLA2G7 group; genes with a green branch belong to the PLA2G4 group; genes with a purple branch belong to the PLA2G3 group; genes with a light blue branch belong to the PLA2G12 group; genes in pink belong to mammals; genes in dark yellow belong to birds; genes in dark green belong to amphibians; genes in sky blue belong to fish. NJ, neighbor-joining; ME, minimum evolution; MP, maximum parsimony.

A total of 49 sequences from 10 species were used to reconstruct a phylogenetic tree for the PLA2 gene family using the NJ, ME, MP methods, as well as the Bayesian information criterion with bootstrap value detection. The details of the included data are presented in Table I. A total of 25 nodes (56.81% in total) showed bootstrap values ≥95% and 34 nodes (77.27% in total) had bootstrap values ≥80% in the Bayes building tree (Fig. 2D). In each subgroup, mammal data, including data from the Sumatran orangutan, pig, Norway rat, human, house mouse, dog and cattle were gathered. The data from the African clawed frog, chicken and zebrafish were much more original than those from mammals, indicating that the taxonomy of host organisms reflects the phylogenetic background of the PLA2 gene family. The vertebrate PLA2 gene family was sorted into nine lineages according to the type of reaction for catalyzing phospholipids. PLA2G7 seems to be the most distant lineage in this gene family, indicating a large number of structural changes accumulating on them. Furthermore, all the groups were divided into two major clades; clade 1 included PLA2G16, PLA2G6, PLA2G10, PLA2G5 and PLA2G15, whereas clade 2 included PLA2G7, PLA2G4, PLA2G3 and PLA2G12. Closer lineages were observed between PLA2G16 and PLA2G6, PLA2G7 and PLA2G4, PLA2G3 and PLA2G12, well as among PLA2G10, PLA2G5 and PLA2G15. Moreover, the phylogenetic relationships obtained by the NJ, ME and MP methods were different (Fig. 2A–C).

### Analysis of positive selection sites of the PLA2 gene family in vertebrates

Positive selection sites were also computed under site, branch and branch-site models for the PLA2 gene family. During site model computing, only P<0.05 and ω>1 indicated the presence of possible positive selection sites. As a result, only M7/M8 met the criteria (P=0.00000, w=2.52322) and showed the following eight positive selection sites: 28N, 34K, 50S, 54T, 58R, 75T, 88Q and 92R (all positive selection sites mentioned in this manuscript refer to amino acids of PLA2G7) (Table II).

Additional calculations were performed to confirm and supplement the results. The branch model was used for positive branch selection. The free-ratio model was significantly higher than the one-ratio model (2ΔlnL=694.2, p=1.306E-93, df=185), indicating heterogeneous selection among branches. Two-ratio models were used using the selected 12 branches; the results revealed that two models (Td and Tf) were significantly different (Pd=3.978E-08, Pf=0.017) at ω>1. Subsequently, branch-site models were used to search for amino acid sites that underwent positive selection in the statistically significant foreground branches Td and Tf (Table III).

Calculation parameters were set as model=2 and Nsite=2 in PAML package version 4.4. The H1 vs. H0 models of the two branches were differed significantly. Eight amino acid sites were found in branch df: 276G, 191K, 327D, 319S, 66G, 67C, 179G and 257Q (Table IV).

**Figure 3:**
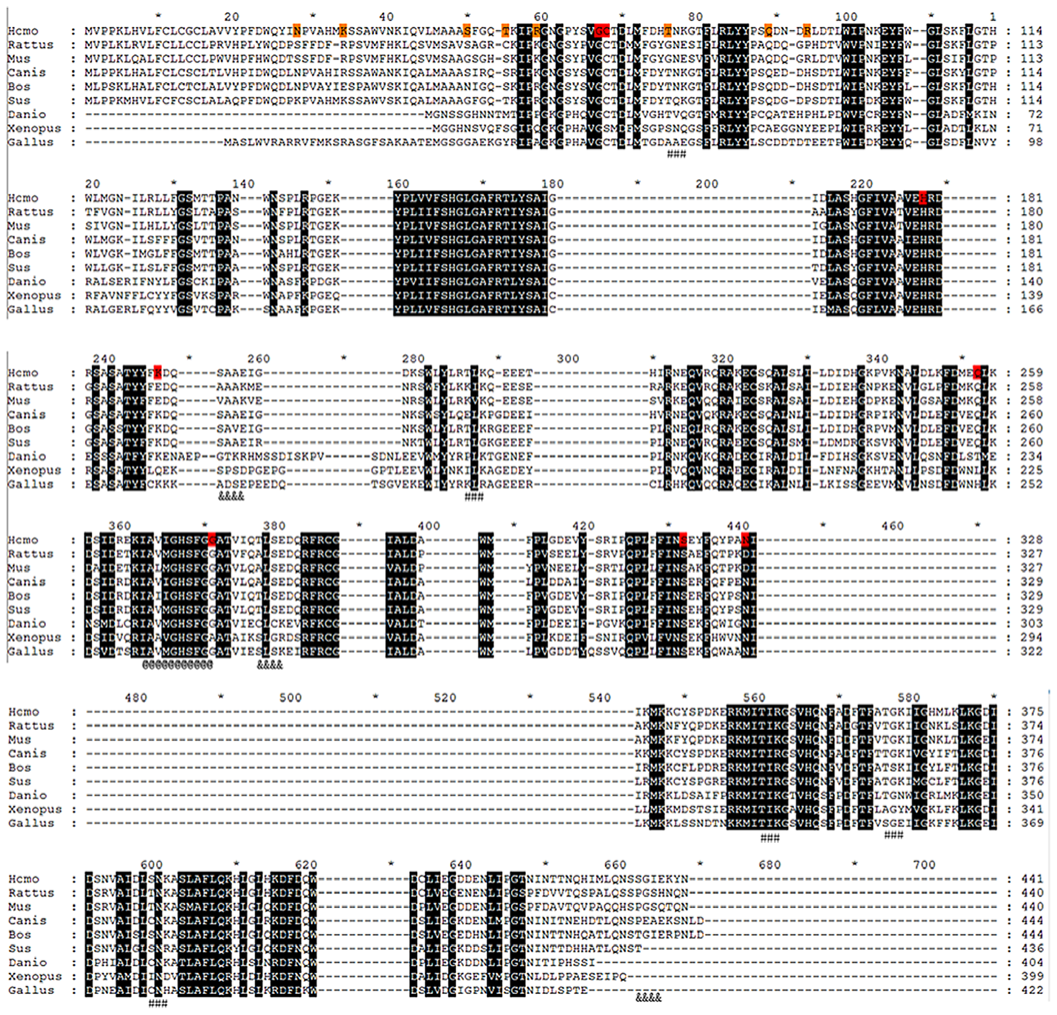
Positive selection sites in planar structure. Amino acid residuals in orange background belong to positive selection sites detected using the site model. Amino acid residues in red background belong to positive selection sites detected using the branch model and branch-site model. #, protein kinase C phosphorylation site; &, casein kinase II phosphorylation site; @, serine active site.

**Figure 4:**
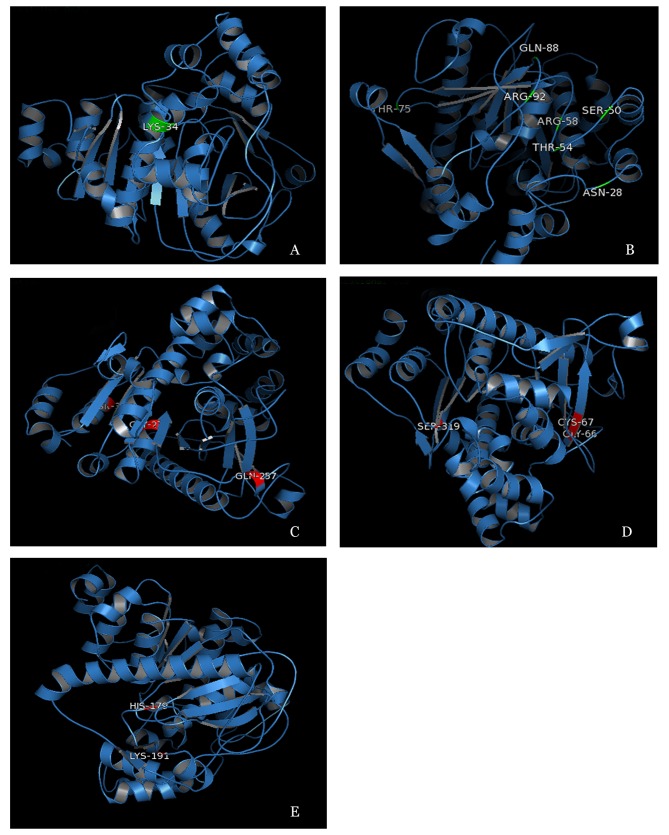
Positive selection sites in three dimensional structures. (A) Positive selection sites from site model on α-helix; (B) Positive selection sites from site model on random coil. (C) Positive selection sites from branch model and branch-site model on α-helix; (D) positive selection sites from branch model and branch-site model on β-sheet; (E) positive selection sites from branch model and branch-site model on random coil.

Using I-TASSER (32–34) (http://zhanglab.ccmb.med.umich.edu/I-TASSER/), four positive selection sites, 327D, 257Q, 276G and 34s, were located in α-helix; three positive selection sites, 66G, 67C and 319S, were located in β-sheet; and nine positive selection sites, 28N, 50S, 54T, 58R, 75T, 88Q, 92R, 179H and 191K, were located in random coil. All details of the positive selection sites are presented in Table V. A planar structure of all positive selection sites is presented in Fig. 3. Positive selection sites, which were detected by site models are three-dimensionally presented in Fig. 4A and B. Positive selection sites, which were detected by branch and branch-site models, are three-dimensionally presented in Fig. 4C–E.

### Distribution of positive selection sites

The functional areas on the AA sequence of PLA2G7_Homo were predicted by PredictProtein. The positive selection site 276G was located in the serine active site, 75T was located in the protein kinase C phosphorylation site and 191K was located near the casein kinase II phosphorylation site.

## Discussion

Available natural and complete sequences of the phospholipase gene family in humans and the PLA2 gene family of vertebrates from the NCBI database were included in the present study. The phospholipase and PLA2 gene families showed different phylogenetic backgrounds and relationships according to the method used for determination (the NJ, ME, MP methods and the Bayesian information criterion). This difference may be attributed to the weakness of these methods. The NJ method focuses on one final topology with branch length estimates, and the observed differences between sequences are inaccurate reflections of the evolutionary distances (35). The construction of an ME tree is time consuming, and examining all topologies is difficult (36). The MP method lacks statistical consistency and does not guarantee the production of a true tree with high probability, given sufficient data (37). Bayesian analysis, which is widely accepted as the most valuable method in phylogenetic analysis and the estimation of positive selection sites, was also employed (26).

The PLA2 family is the most complex gene family of phospholipases (38,39). The majority of PLA2 genes encode secreted enzymes with physiological features involved in catalyzing platelet activity (40), controlling lipid metabolism (2) and mediating inflammations (5). The dysfunction of these genes may lead to stroke (14).

The PLA2G7 coding enzyme, Lp-PLA2, has attracted considerable attention due to its crucial function in platelet gathering in cardiovascular and cerebrovascular diseases (11). Lp-PLA2 is a new biological marker for detecting vasculitis (41). Unlike multiple clinical trials and diagnostic estimations of Lp-PLA2 mass and activity in the circulation (18), data on the phylogenetic background of the PLA2 gene family and the positive selection of amino acid residues on PLA2G7 genes are limited (42–44).

According to the PLA2 phylogenetic tree built using the Bayesian information criterion, PLA2G7 is one of the most evolutionarily distant members of PLA2 proteins, an indication of a fast-evolving lineage with numerous structural changes. Moreover, lineage-specific expansion and divergence events were not observed from low-order to high-order vertebrates. The first duplication of the PLA2G7 group led to the emergence of lineages in the Norway rat and the house mouse, and the residual mammals shared duplication with birds, fish and amphibians. Thus, at least two duplications are present in mammals. Moreover, the PLA2G4 family presented the closest lineage to the PLA2G7 family, indicating that PLA2G4 may be another gene that mediates platelet gathering.

In the present study, we identified specific amino acid residues of PLA2G7, which are targets of positive selection. According to the site model result, eight positive selection sites, 28N, 34K, 50S, 54T, 58R, 75T, 88Q and 92R, were found, and eight amino acid sites, 276G, 191K, 327D, 319S, 66G, 67C, 179G and 257Q, were found by the branch and branch-site models. No identical positive selection sites were found among the site, branch and branch-site models.

Functional structure, the protein kinase C phosphorylation site (45), the casein kinase II phosphorylation site (46) and the serine active site (47) were widely scattered along the PLA2G7 peptide chain. The serine active site is a conserved region centered on a serine residue and has the function of catalyzing fatty acid transfer between phosphatidylcholine and cholesterol. According to the Bayesian analysis, a positive selection site, 276G, was located on serine active region, indicating its similar function. It has been previoulsy demonstrated that Lp-PLA2 mediates atherosclerosis by promoting platelet gathering and adherence to vessels (48) and a previous study (49) suggests that, apart from promoting platelet gathering, Lp-PLA2 may also alter cholesterol metabolism in atherosclerosis. Protein kinase C can modify the function of a protein by increasing or decreasing the protein's activity, stabilizing it or marking it for destruction. The positive selection site, 75T, located on the protein kinase C phosphorylation site, indicated its function on altering Lp-PLA2 activity. Casein kinase II is a protein kinase that phosphorylates many different proteins and is relevant to changes in macrophage gene expression during atherosclerosis (50). We found that 191K was located near the casein kinase II phosphorylation site, indicating that Lp-PLA2 may also increase macrophage gene expression in atherosclerosis. However, further validation of such sites is required in order to obtain richer experimental data.

In conclusion, the PLA2 gene family is the most complex gene family among the phospholipases. A number of studies, including clinical trials have focused on the diagnostic estimation of the mass and activity of PLA2 coding enzymes in the circulation (18,51,52); however, phylogenetic analyses of the PLA2 gene family and positive selection amino acid residues on PLA2G7 genes are limited. The present study focused on the phospholipase and PLA2 gene families employing phylogenetic analysis using the NJ, ME and MP methods, as well as the Bayesian information criterion. Positive selection sites were detected for the PLA2 family using site, branch and branch-site models. A total of 49 sequences from 10 different species were selected for the analysis. Phylogenetic analysis of the PLA2 gene family in vertebrates suggests that PLA2G5 is the origin of this gene family, and that PLA2G7 is one of the most evolutionarily distant PLA2 proteins. Eight positive selection sites were detected using the site model, whereas eight positive selection sites were detected using the branch and branch-site models.

## Figures and Tables

**Table I:** Data on PLA2 subfamily in vertebrates.

Abb	Species	Country	Year	NCBI-PID	NCBI-GID	Chromosome	Taxonomic groups
PLA2G7_Xenopus	African clawed frog	USA	2002	NP_001017267.1	NM_001017267.2	Un	Amphibians
PLA2G7_Sus	Pig	Belgium	2009	NP_001106484.1	NM_001113013.1	7	Mammals
PLA2G7_Rattus	Norway rat	Sweden	2012	NP_001009353.1	NM_001009353.1	9	Mammals
PLA2G7_Mus	House mouse	China	2013	NP_038765.2	NM_013737.5	17	Mammals
PLA2G7_Homo	Human	China	2013	NP_001161829.1	NM_001168357.1	6	Mammals
PLA2G7_Gallus	Chicken	USA	1995	NP_990300.1	NM_204969.1	3	Birds
PLA2G7_Danio	Zebrafish	China	2004	NP_998354.1	NM_213189.1	20	Fish
PLA2G7_Canis	Dog	USA	1995	NP_001003198.1	NM_001003198.1	12	Mammals
PLA2G7_Bos	Cattle	USA	2009	NP_777003.2	NM_174578.4	23	Mammals
PLA2G6_Xenopus	African clawed frog	USA	2002	NP_001072661.1	NM_001079193.1	Un	Amphibians
PLA2G6_Rattus	Norway rat	Germany	2012	NP_001005560.1	NM_001005560.1	7	Mammals
PLA2G6_Mus	House mouse	Japan	2013	NP_001185954.1	NM_001199025.1	15	Mammals
PLA2G6_Homo	Human	Brazil	2013	NP_003551.2	NM_003560.2	22	Mammals
PLA2G6_Gallus	Chicken	Austria	2008	NP_001124210.1	NM_001130738.1	1	Birds
PLA2G5_Rattus	Norway rat	UK	2004	NP_058870.1	NM_017174.1	5	Mammals
PLA2G5_Mus	House mouse	USA	2013	NP_001116426.1	NM_001122954.1	4	Mammals
PLA2G5_Homo	Human	USA	2013	NP_000920.1	NM_000929.2	1	Mammals
PLA2G5_Gallus	Chicken	-	-	NP_001264973.1	NM_001278044.1	21	Birds
PLA2G5_Bos	Cattle	USA	2009	NP_001179981.1	NM_001193052.1	2	Mammals
PLA2G4_Xenopus	African clawed frog	USA	2002	NP_001080867.1	NM_001087398.1	Un	Amphibians
PLA2G4_Rattus	Norway rat	USA	2011	NP_598235.2	NM_133551.2	13	Mammals
PLA2G4_Pongo	Sumatran orangutan	-	-	NM_001132692.1	NP_001126164.1	1	Mammals
PLA2G4_Mus	House mouse	Japan	2013	NP_032895.1	NM_008869.3	1	Mammals
PLA2G4_Homo	Human	Japan	2013	NP_077734.1	NM_024420.2	1	Mammals
PLA2G4_Danio	Zebrafish	USA	2013	NP_571370.1	NM_131295.2	2	Fish
PLA2G3_Rattus	Norway rat	Singapore	2013	NP_001099485.1	NM_001106015.1	14	Mammals
PLA2G3_Mus	House mouse	Japan	2013	NP_766379.2	NM_172791.2	11	Mammals
PLA2G3_Homo	Human	Spain	2013	NP_056530.2	NM_015715.3	22	Mammals
PLA2G3_Bos	Cattle	USA	2009	NP_001074379.1	NM_001080910.1	17	Mammals
PLA2G16_Sus	Pig	Japan	2007	NP_001231443.1	NM_001244514.1	2	Mammals
PLA2G16_Rattus	Norway rat	France	2001	NP_058756.2	NM_017060.2	1	Mammals
PLA2G16_Mus	House mouse	Japan	2012	NP_644675.2	NM_139269.2	19	Mammals
PLA2G16_Homo	Human	USA	2012	NP_009000.2	NM_007069.3	11	Mammals
PLA2G16_Bos	Cattle	China	2012	NP_001068748.1	NM_001075280.2	29	Mammals
PLA2G15_Xenopus	African clawed frog	USA	2002	NP_001089365.1	NM_001095896.1	Un	Amphibians
PLA2G15_Rattus	Norway rat	Japan	2005	NP_001004277.1	NM_001004277.2	19	Mammals
PLA2G15_Mus	House mouse	USA	2013	NP_598553.1	NM_133792.2	8	Mammals
PLA2G15_Homo	Human	Canada	2010	NP_036452.1	NM_012320.3	16	Mammals
PLA2G15_Canis	Dog	USA	2007	NP_001002940.1	NM_001002940.1	5	Mammals
PLA2G15_Bos	Cattle	USA	2009	NP_776985.2	NM_174560.2	18	Mammals
PLA2G12_Xenopus	African clawed frog	USA	2003	NP_001017096.1	NM_001017096.2	Un	Amphibians
PLA2G12_Sus	Pig	Japan	2007	NP_001230267.1	NM_001243338.1	14	Mammals
PLA2G12_Rattus	Norway rat	USA	2002	NP_001102035.1	NM_001108565.1	2	Mammals
PLA2G12_Mus	House mouse	Italy	2011	NP_075685.2	NM_023196.4	3	Mammals
PLA2G12_Homo	Human	Canada	2010	NP_110448.2	NM_030821.4	4	Mammals
PLA2G10_Rattus	Norway rat	France	1999	NP_058872.1	NM_017176.2	10	Mammals
PLA2G10_Mus	House mouse	Japan	2013	NP_036117.1	NM_011987.2	16	Mammals
PLA2G10_Homo	Human	Iran	2013	NP_003552.1	NM_003561.1	16	Mammals
PLA2G10_Gallus	Chicken	-	-	NP_001171686.1	NM_001184757.1	14	Birds

Abb, abbreviation; NCBI-PID, protein ID in NCBI; NCBI-GID, gene ID in NCBI.

**Table II:** Parameter estimates and likelihood scores of PLA2 for site models in PAML.

Model	np	Estimates of parameters	lnL	LRT pairs	df	2ΔlnL	p-value	Positively selected sites BEB (%)
M0:one ratio	94	ω=0.16513	−41914.67	M0/M3	4	366.4 **5.04128E-78**		None
M3:discrete	98	p_0_=0.18381, p_1_=0.61322, p_2_=0.20297, ω_0_=0.06840 ω_1_=0.14537,ω_2_=0.42278	−41731.47					
M1a:neutral	95	p_0_=0.92349, p_1_=0.07651,ω_0_=0.15503, ω_1_=1.00000	−41830.79	M1a/M2a	2	0	1.00000	33M (55.5), 34K (52.8)
M2a:selection	97	p_0_=0.92349, p_1_=0.03595, p_2_=0.04056, ω_0_=0.15503, ω_1_=1.00000, ω_2_=1.00000	−41830.79					
M7:β	95	p=2.25732, q=10.02539	−45800.80	M7/M8	2	8103.86	**0.00000**	28N (65.8), 34K (53.7), 50S (60.4), 54T (64.6), 58R (64.9), 75T (70.0), 88Q (77.2), 92R (50.1)
M8:β and ω	97	p_0_=0.99999, p=0.17653, q=1.31411, p_1_=0.00001, ω=2.52322	−41748.87					

Selection analysis by site models was performed using CODEML implemented in PAML. np, number of free parameters; lnL, loglikelihood; LRT, likelihood ratio test; df, degrees of freedom; 2ΔlnL, twice the log-likelihood difference of the models compared; BEB, Bayes empirical Bayes approach. p-values with bold font indicate statistical significance.

**Table III:** Parameter estimates and likelihood scores of PLA2 for site models in PAML parameter estimates and likelihood scores of PLA2 for branch models in PAML.

Model	np	Estimates of parameters	lnL	LRT pairs	df	2ΔlnL	p-value
Fr:free ratios	185		−41567.57	M0/Fr	91	694.2	**1.306E-93**
Tx:two ratios							
Ta	95	ω_0_=0.1649, **ω_a_=3.6122**	−41913.33	M0/Ta	1	2.68	0.102
Tb	95	ω_0_=0.1645, **ω_b_=49.9559**	−41914.34	M0/Tb	1	0.66	0.417
Tc	95	ω_0_=0.1651, **ω_c_=21.4714**	−41917.97	M0/Tc	1	6.6	0.010
Td	95	ω_0_=0.1594, **ω_d_=37.8875**	−41899.59	M0/Td	1	30.16	**3.978E-08**
Te	95	ω_0_=0.1652, **ω_e_=59.5984**	−41914.39	M0/Te	1	0.56	0.454
Tf	95	ω_0_=0.1666, **ω_f_=87.3449**	−41911.84	M0/Tf	1	5.66	**0.017**
Tg	95	ω_0_=0.1656, **ω_g_=51.0797**	−41914.47	M0/Tg	1	0.4	0.527
Th	95	ω_0_=0.1650, **ω_h_=34.3683**	−41914.62	M0/Th	1	0.1	0.752
Ti	95	ω_0_=0.1653, **ω_i_=49.6517**	−41914.51	M0/Ti	1	0.3	0.584
Tj	95	ω_0_=0.1650, **ω_j_=37.3306**	−41914.52	M0/Tj	1	0.3	0.584
Tk	95	ω_0_=0.1650, **ω_k_=21.5084**	−41914.66	M0/Tk	1	0.02	0.888
Tl	95	ω_0_=0.1655, **ω_l_=24.465**1	−41914.55	M0/Tl	1	0.24	0.624

Selection analysis by branch models was performed using CODEML implemented in PAML. np, number of free parameters; lnL, loglikelihood; LRT, likelihood ratio test; df, degrees of freedom; 2ΔlnL, twice the log-likelihood difference of the models compared. p-values with bold font indicate statistical significance.

**Table IV:** Parameter estimates and likelihood scores of PLA2 for branch-site models in PAML.

Model	np	Estimates of parameters	lnL	LRT pairs	df	2ΔlnL	p-value	Positively selected sites BEB (%)
BSa1	97	p_0_=0.78310, p_1_=0.05618, p_2a_=0.14996, p_2b_=0.01076, ω_0_=0.15077, ω_1_=1.00000, b:ω_2a_=0.15077, ω_2b_=1.00000, f:ω_2a_=999.00000, ω_2b_=999.00000	−41811.53	BSa1/BSa0-fix	1	17.62	2.70E-05	276G (61.7) 191K (58.8) 327D (58.7) 319S (58.2)
BSa0-fix	96	p_0_=0.64515, p_1_=0.04724, p_2a_=0.28662, p_2b_=0.02099 ω_0_=0.15015, ω_1_=1.00000, b:ω_2a_=0.15015, ω_2b_=0.15015, f:ω_2a_=1.00000, ω_2b_=1.00000	−41820.34					
BSb1	97	p_0_=0.89293, p_1_=0.07409, p_2a_=0.03045, p_2b_=0.00253, w_0_=0.15484, ω_1_=1.00000, b:ω_2a_=0.15484, ω_2b_=1.00000, f:ω_2a_=111.98807, ω_2b_=111.98807	−41828.90	BSb1/BSb0-fix	1	3.34	6.76E-02	66G (88.2) 67C (89.7) 179H (75.9) 257Q (58.0)
BSb0-fix	96	p_0_=0.89547, p_1_=0.07423, p_2a_=0.02798, p_2b_=0.00232, w_0_=0.15479, ω_1_=1.00000, b:ω_2a_=0.15479, ω_2b_=1.00000, f:ω_2a_=1.00000, ω_2b_=1.00000	−41830.57					

Selection analysis by branch-site models was performed using codeml implemented in PAML. BS, branch-site; np, number of free parameters; lnL, loglikelihood; LRT, likelihood ratio test; df, degrees of freedom; 2ΔlnL, twice the log-likelihood difference of the models compared. BEB, Bayes empirical Bayes approach.

**Table V:** Positive selection sites by site model, branch model and site-branch model.

Location	Amino acid	Secondary struscture	Posterior probability (%)	Model
28	Asn	Random coil	65.8	Site model
34	Lys	α-helix	53.7	Site model
50	Ser	Random coil	60.4	Site model
54	Thr	Random coil	64.6	Site model
58	Arg	Random coil	64.9	Site model
66	Gly	β-pleated sheet	88.2	Branch model and branch-site model
67	Cys	β-pleated sheet	89.7	Branch model and branch-site model
75	Thr	Random coil	70.0	Site model
88	Gln	Random coil	77.2	Site model
92	Arg	Random coil	50.1	Site model
179	His	Random coil	75.9	Branch model and branch-site model
191	Lys	Random coil	58.8	Branch model and branch-site model
257	Gln	α-helix	58.0	Branch model and branch-site model
276	Gly	α-helix	61.7	Branch model and branch-site model
319	Ser	β-pleated sheet	58.2	Branch model and branch-site model
327	Asn	α-helix	58.7	Branch model and branch-site model

All positive-selection sites were marked on AA sequence of PLA2G7_Homo.

## References

[1] Heinrikson RL, Krueger ET, Keim PS (1977). Amino acid sequence of phospholipase A2-alpha from the venom of Crotalus adamanteus. A new classification of phospholipases A2 based upon structural determinants. J Biol Chem.

[2] de Beer FC, Webb NR (2006). Inflammation and atherosclerosis: Group IIa and Group V sPLA2 are not redundant. Arterioscler Thromb Vasc Biol.

[3] Starkl P, Marichal T, Galli SJ (2013). PLA2G3 promotes mast cell maturation and function. Nat Immunol.

[4] Mackenzie LS, Lymn JS, Hughes AD (1833). Linking phospholipase C isoforms with differentiation function in human vascular smooth muscle cells. Biochim Biophys Acta.

[5] Clark JD, Schievella AR, Nalefski EA, Lin LL (1995). Cytosolic phospholipase A2. J Lipid Mediat Cell Signal.

[6] Johansson P, Thesleff S (1968). A comparison of the effects of phos-pholipase C and tetrodotoxin on spike generation in muscle. Eur J Pharmacol.

[7] Jackson TN, Sunagar K, Undheim EA (2013). Venom down under: dynamic evolution of Australian elapid snake toxins. Toxins (Basel).

[8] Margres MJ, Aronow K, Loyacano J, Rokyta DR The venom-gland transcriptome of the eastern coral snake (Micrurus fulvius) reveals high venom complexity in the intragenomic evolution of venoms. BMC Genomics.

[9] Dennis EA (1994). Diversity of group types, regulation, and function of phospholipase A2. J Biol Chem.

[10] Smith AD, Winkler H (1968). Lysosomal phospholipases A1 and A2 of bovine adrenal medulla. Biochem J.

[11] Pan YH, Yu BZ, Berg OG, Jain MK, Bahnson BJ (2002). Crystal structure of phospholipase A2 complex with the hydrolysis products of platelet activating factor: equilibrium binding of fatty acid and lysophospholipid-ether at the active site may be mutually exclusive. Biochemistry.

[12] Ohtsuki T, Watanabe H, Toru M, Arinami T (2002). Lack of evidence for associations between plasma platelet-activating factor acetyl-hydrolase deficiency and schizophrenia. Psychiatry Res.

[13] Hixon MS, Ball A, Gelb MH (1998). Calcium-dependent and -independent interfacial binding and catalysis of cytosolic group IV phospholipase A2. Biochemistry.

[14] Retraction: CDP-choline significantly restores phosphatidylcholine levels by differentially affecting phospholipase A2 and CTP: phosphocholine cytidylyltransferase after stroke. J Biol Chem.

[15] Fitzpatrick AL, Irizarry MC, Cushman M, Jenny NS, Chi GC, Koro C (2014). Lipoprotein-associated phospholipase A2 and risk of dementia in the Cardiovascular Health Study. Atherosclerosis.

[16] Gui YX, Xu ZP, Wen-Lv Q, Liu HM, Zhao JJ, Hu XY (2013). Four novel rare mutations of PLA2G6 in Chinese population with Parkinson's disease. Parkinsonism Relat Disord.

[17] Farooqui AA, Ong WY, Horrocks LA (2006). Inhibitors of brain phospholipase A2 activity: their neuropharmacological effects and therapeutic importance for the treatment of neurologic disorders. Pharmacol Rev.

[18] Holmes MV, Simon T, Exeter HJ (2013). Secretory phospholipase A(2)-IIA and cardiovascular disease: a mendelian randomization study. J Am Coll Cardiol.

[19] Ohno M, Menez R, Ogawa T (1998). Molecular evolution of snake toxins: is the functional diversity of snake toxins associated with a mechanism of accelerated evolution?. Prog Nucleic Acid Res Mol Biol.

[20] Edgar RC MUSCLE: a multiple sequence alignment method with reduced time and space complexity. BMC Bioinformatics.

[21] Suyama M, Torrents D, Bork P (2006). PAL2NAL: robust conversion of protein sequence alignments into the corresponding codon alignments. Nucleic Acids Res.

[22] Kumar S, Tamura K, Nei M (2004). MEGA3: Integrated software for Molecular Evolutionary Genetics Analysis and sequence alignment. Brief Bioinform.

[23] Posada D (2003). Using MODELTEST and PAUP^*^ to select a model of nucleotide substitution. Curr Protoc Bioinformatics.

[24] Guindon S, Gascuel O (2003). A simple, fast, and accurate algorithm to estimate large phylogenies by maximum likelihood. Syst Biol.

[25] Posada D, Crandall KA (1998). MODELTEST: testing the model of DNA substitution. Bioinformatics.

[26] Ronquist F, Huelsenbeck JP (2003). MrBayes 3: Bayesian phylogenetic inference under mixed models. Bioinformatics.

[27] Huelsenbeck JP, Ronquist F (2001). MRBAYES: Bayesian inference of phylogenetic trees. Bioinformatics.

[28] Yang Z (2007). PAML 4: phylogenetic analysis by maximum likelihood. Mol Biol Evol.

[29] Yang Z, Wong WS, Nielsen R (2005). Bayes empirical bayes inference of amino acid sites under positive selection. Mol Biol Evol.

[30] Nielsen R, Yang Z (1998). Likelihood models for detecting positively selected amino acid sites and applications to the HIV-1 envelope gene. Genetics.

[31] Rost B, Yachdav G, Liu J (2004). The PredictProtein server. Nucleic Acids Res.

[32] Roy A, Yang J, Zhang Y (2012). COFACTOR: an accurate comparative algorithm for structure-based protein function annotation. Nucleic Acids Res.

[33] Roy A, Kucukural A, Zhang Y (2010). I-TASSER: a unified platform for automated protein structure and function prediction. Nat Protoc.

[34] Zhang Y I-TASSER server for protein 3D structure prediction. BMC Bioinformatics.

[35] Saitou N, Nei M (1987). The neighbor-joining method: a new method for reconstructing phylogenetic trees. Mol Biol Evol.

[36] Thompson EA (1973). The method of minimum evolution. Ann Hum Genet.

[37] Goodman M, Moore GW, Barnabas J, Matsuda G (1974). The phylogeny of human globin genes investigated by the maximum parsimony method. J Mol Evol.

[38] Xin H, Chen ZY, Lv XB, Liu S, Lian ZX, Cai SL (2013). Serum secretory phospholipase A2-IIa (sPLA2-IIA) levels in patients surviving acute myocardial infarction. Eur Rev Med Pharmacol Sci.

[39] Sullivan AH (1985). A measurement of the local energy deposition by antiprotons coming to rest in tissue-like material. Phys Med Biol.

[40] Duivenvoorden R, Mani V, Woodward M (2013). Relationship of serum inflammatory biomarkers with plaque inflammation assessed by FDG PET/CT: the dal-PLAQUE study. JACC Cardiovasc Imaging.

[41] Mangili A, Ahmad R, Wolfert RL (2014). Lipoprotein-associated phospholipase A2, a novel cardiovascular inflammatory marker, in HIV-infected patients. Clin Infect Dis.

[42] Hariprasad G, Srinivasan A, Singh R (2013). Structural and phylogenetic basis for the classification of group III phospholipase A2. J Mol Model.

[43] Magrioti V, Kokotos G (2010). Phospholipase A2 inhibitors as potential therapeutic agents for the treatment of inflammatory diseases. Expert Opin Ther Pat.

[44] Gibbs HL, Rossiter W (2008). Rapid evolution by positive selection and gene gain and loss: PLA_2_ venom genes in closely related Sistrurus rattlesnakes with divergent diets. J Mol Evol.

[45] Woodgett JR, Gould KL, Hunter T (1986). Substrate specificity of protein kinase C. Use of synthetic peptides corresponding to physiological sites as probes for substrate recognition requirements. Eur J Biochem.

[46] Pinna LA (1990). Casein kinase 2: an ‘eminence grise’ in cellular regulation?. Biochim Biophys Acta.

[47] Chapus C, Rovery M, Sarda L, Verger R (1988). Minireview on pancreatic lipase and colipase. Biochimie.

[48] Caslake MJ, Packard CJ (2003). Lipoprotein-associated phospholipase A2 (platelet-activating factor acetylhydrolase) and cardiovascular disease. Curr Opin Lipidol.

[49] Feng LM, Feng GF, Chen Y Evaluation of lipoprotein-associated phospholipase A2 in healthy Chinese Han adult serum. Lipids Health Dis.

[50] Harvey EJ, Li N, Ramji DP (2007). Critical role for casein kinase 2 and phosphoinositide-3-kinase in the interferon-gamma-induced expression of monocyte chemoattractant protein-1 and other key genes implicated in atherosclerosis. Arterioscler Thromb Vasc Biol.

[51] Tai W, Garcia M, Mlynash M, Kemp S, Albers GW, Olivot JM (2014). Lipoprotein phospholipase A2 mass and activity are not associated with the diagnosis of acute brain ischemia. Cerebrovasc Dis.

[52] Nozadze DN, Sergienko IV, Balakhonova TV, Semenova AE, Vlasik TN, Kukharchuk VV (2014). Lipoprotein-associated phospholipase A2 serum levels in patients from different categories of cardiovascular risk. Kardiologiia.

